# In Vivo Localization of the Human Velocity Storage Mechanism and Its Core Cerebellar Networks by Means of Galvanic-Vestibular Afternystagmus and fMRI

**DOI:** 10.1007/s12311-022-01374-8

**Published:** 2022-02-25

**Authors:** Maxine Rühl, Rebecca Kimmel, Matthias Ertl, Julian Conrad, Peter zu Eulenburg

**Affiliations:** 1 Department of Neurology, University Hospital Munich, Ludwig Maximilians University, Munich, Germany; 2 German Center for Vertigo and Balance Disorders, University Hospital Munich, Ludwig Maximilians University, Munich, Germany; 3 Department of Psychology, University of Bern, Bern, Switzerland; 4 Institute for Neuroradiology, University Hospital Munich, Ludwig Maximilians University, Munich, Germany

**Keywords:** Velocity storage mechanism, Galvanic vestibular stimulation, Cerebellum, Uvula, Neuroimaging

## Abstract

Humans are able to estimate head movements accurately despite the short half-life of information coming from our inner ear motion sensors. The observation that the central angular velocity estimate outlives the decaying signal of the semicircular canal afferents led to the concept of a velocity storage mechanism (VSM). The VSM can be activated via visual and vestibular modalities and becomes manifest in ocular motor responses after sustained stimulation like whole-body rotations, optokinetic or galvanic vestibular stimulation (GVS). The VSM has been the focus of many computational modelling approaches; little attention though has been paid to discover its actual structural correlates. Animal studies localized the VSM in the medial and superior vestibular nuclei. A significant modulation by cerebellar circuitries including the uvula and nodulus has been proposed. Nevertheless, the corresponding neuroanatomical structures in humans have not been identified so far. The aim of the present study was to delineate the neural substrates of the VSM using high-resolution infratentorial fMRI with a fast T2* sequence optimized for infratentorial neuroimaging and via video-oculography (VOG). The neuroimaging experiment (*n*=20) gave first in vivo evidence for an involvement of the vestibular nuclei in the VSM and substantiate a crucial role for cerebellar circuitries. Our results emphasize the importance of cerebellar feedback loops in VSM most likely represented by signal increases in vestibulo-cerebellar hubs like the uvula and nodulus and lobule VIIIA. The delineated activation maps give new insights regarding the function and embedment of Crus I, Crus II, and lobule VII and VIII in the human vestibular system.

**Supplementary Information:**

The online version contains supplementary material available at 10.1007/s12311-022-01374-8.

## Introduction

After sustained viewing of optokinetic stimuli or rotation in darkness, a decaying nystagmus can be observed when subjects are placed in total darkness. The so-called optokinetic afternystagmus (OKAN) and the post rotatory nystagmus have been interpreted as motor responses to an extended central excitatory state and are thought to be mediated by the velocity storage mechanism (VSM) [1–3]. Traditionally, the velocity storage integrator was mainly considered a mechanism to optimize performance during low-frequency vestibular stimulation by transforming the short time constant response at the semicircular canal afferents to a prolonged time constant [4, 5]. Recently, this view has been challenged by mathematical models using Bayesian inference, which suggested a pivotal role of the VSM as a multisensory integrator and rotation estimator allowing for an optimal multisensory processing during mid-frequency rotation [6]. These new models highlighted the importance of “velocity feedback loops” interacting with the VSM to improve the rotation estimate by adjusting for mismatches between the internal estimate of rotation and head motion relative to gravity [6].

Animal studies suggested that the VSM might be coded by neurons in the nucleus prepositus hypoglossi and the medial and superior vestibular nucleus [7–10]. The medial and superior vestibular nuclei contain vestibular-only (VO) and vestibular plus saccade (VPS) canal-related neurons. The time constants and sensitivities of activity to eye velocity during different modalities of stimuli measured in single unit recording suggested a role of these neurons in transmitting eye velocity signals related to the VSM [5]. On the other hand, inhibitory cerebellar circuitries involving the nodulus and the uvula seem to be closely linked with the velocity storage mechanism, possibly acting as a central “dumping mechanism” [11]. In line with these findings, a recent study showed a reduced time constant for the post rotatory nystagmus after intake of GABA_B_ agonists in humans [12, 13]. However, the structural correlates of the velocity storage in humans have never been localized.

Analogous to the ocular motor responses after sustained rotation and optokinetic stimulation, an afternystagmus has been observed after prolonged galvanic vestibular stimulation (GVS), which has been ascribed to the velocity storage mechanism [14, 15]. Transmastoideal GVS is a robust vestibular stimulus recruiting both hair cells and vestibular afferent fibers by means of current and thereby modulates the tonic firing rate of the vestibular afferents [16–18]. As it may mimic different types of motion in the absence of actual head motion, it is the ideal stimulus for neuroimaging studies [19, 20]. GVS elicits repeatable ocular motor responses with a predominant torsional and a horizontal component, with the upper side of the bulbus rotating away from the stimulated side to the negative electrode [14, 20]. In contrast to the ocular motor responses to GVS, only few studies described the subsequent afternystagmus in more detail [14, 21, 22]. Here, a reversal of nystagmus direction at the offset of GVS has been observed, with dynamics probably modulated by the duration and current intensity of the preceding GVS [14]. These studies investigated either near-threshold current intensities of 0.1–0.9 mA or high intensities of 5 mA and stimulus duration up to 5 min. These stimulation patterns are neither feasible nor applicable to produce robust effects in fMRI experiments. They are mostly not tolerable by subjects due to the number of repetitions needed for a robust effect size and the concurrent interfering side effects, which in return might mask the actual brain activation maps of interest [23].

In this study, we aimed to localize the driving mechanism of the GVS-induced afternystagmus, the velocity storage integrator, by means of high-resolution infratentorial neuroimaging and video-oculography. Firstly, we aimed to characterize the afternystagmus using GVS and video-oculography in order to identify a robust and feasible GVS stimulation pattern. In a second step, we used the optimal derived stimulus in neuroimaging to evoke a replicable afternystagmus and thereby depict the neuroanatomical correlates of the human velocity storage mechanism in vivo at the highest spatio-temporal resolution.

## Material and Methods

### Participants

Thirty healthy volunteers (15 female, 15 male) with a mean age of 27 years (range: 19–37 years) participated in the video-oculography baseline experiment outside the scanner, twenty of which (11 female, 9 male, mean age of 26.3 years) then could be recruited for the subsequent neuroimaging sessions. All participants gave their written and informed consent. The modified laterality quotient of handedness and footedness according to the 14-item inventory of the Edinburgh test [24] was determined. All subjects were right-handed, with normal uncorrected vision, and binocular parity. In the study, only subjects without a previous history of neurotological or ocular motor disorder and without regular medication were included. All subjects underwent a detailed neuro-otological examination before the experiments to exclude possible latent pathologies of the inner ear. The study was approved by the local Ethics Committee and in accordance with the Declaration of Helsinki (2013). Subjects were paid for participation. We followed the guidelines and principles for reporting fMRI studies laid down earlier by Poldrack and colleagues [25].

### GVS Video-Oculography Experiment

**Fig. 1:**
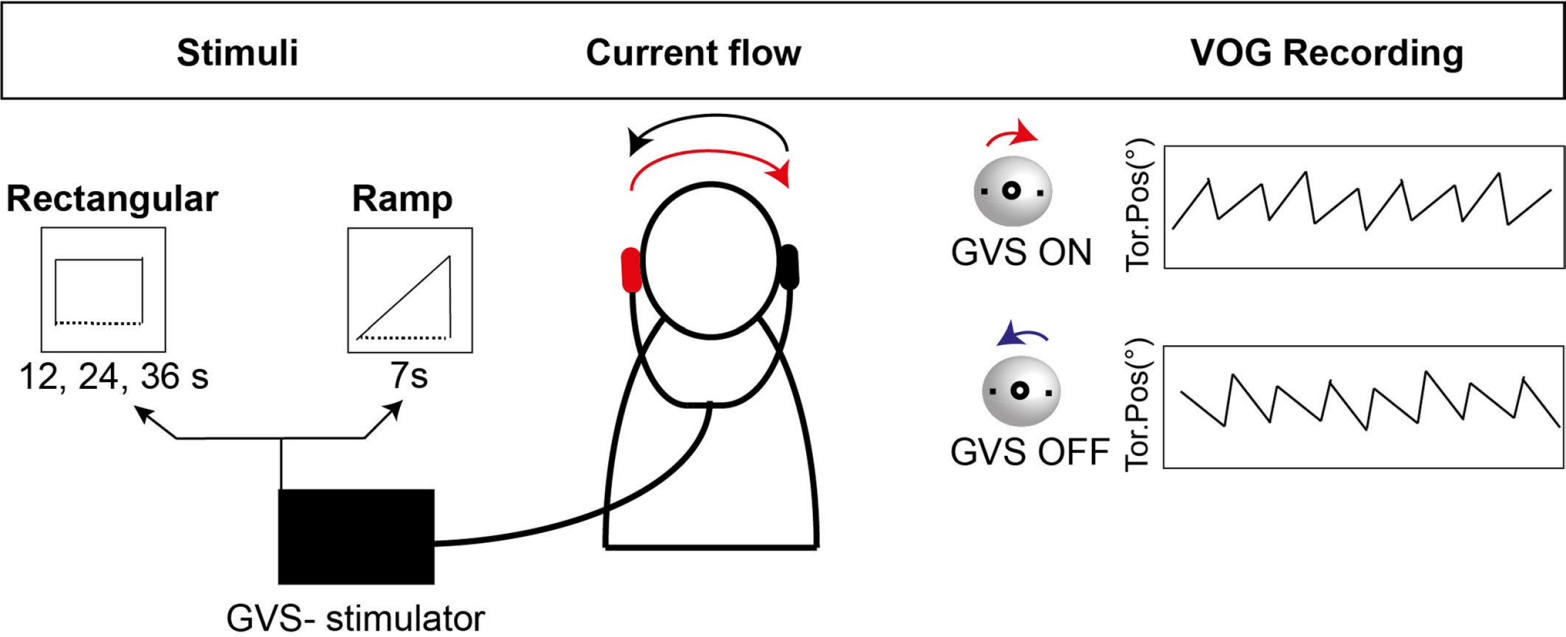
**Experimental protocol for GVS stimulation and concurrent video-oculography.** GVS was applied via electrodes on each mastoid. Stimulation patterns consisted of either rectangular stimuli of 12-, 24-, or 36-s duration with a fixed intertrial interval or ramp stimuli of 6 s with varying interstimulus intervals. Torsional eye movements were recorded using an infrared VOG setup with scleral markers. During GVS, the quick phase of the elicited nystagmus rotates away from the stimulated side, whereas immediately at GVS offset, the afternystagmus reverses direction

Two experiments using two different GVS stimuli were conducted to determine the optimal stimulus to evoke a robust afternystagmus in the ensuing fMRI experiment. Twenty-one subjects were examined in the first experiment, and 10 subjects in the second experiment. Data from two subjects had to be discarded in the second experiment due to insufficient scleral markers. The thirty subjects were examined using an infrared video-oculography system (EyeSeeCam, Germany) [26] outside the scanner on a separate day before the fMRI experiment. Calibration of the VOG system was performed before conducting the experiments as implemented in the EyeSeeCam software. GVS was applied via bimastoidal electrodes (3 mA current intensity) using a custom-made GVS stimulator after local anesthesia of the mastoid [23] while subjects were lying in a supine position similar to the position in the scanner. Scleral markers were applied to measure ocular torsion reliably. The first experiment focused on the occurrence of afternystagmus after ramp stimuli and the dependency of its duration on the duration of the intertrial intervals. Unilateral ramp stimuli were applied at each mastoid (3 mA, ramp on-set duration 7 s, no stimulus plateau, 64 stimulation cycles, total duration of experiment 33.4 min) with intertrial intervals with varying durations [6, 9, 12, 24, 60] presented in a pseudo-randomized order (Fig. 1). Eye movement recording was started 60 s before the experiment started, in order to display the eye movements in rest and test for a physiological spontaneous upbeat-nystagmus [27].

In the second experiment, we aimed at studying the effect of stimulus duration on the duration of the afternystagmus elicited by rectangular stimuli. Here, bilateral rectangular stimuli (3 mA) of either 12-, 24-, or 36-s duration (30 stimulation cycles, total duration of experiment 30 min) with a fixed intertrial interval of 38 s were applied in a pseudo-randomized order. After the stimulation, subjects had to evaluate the perceived sensations during GVS via a standardized questionnaire. All subjects perceived motion during stimulation. The statistical analysis of the data was performed using SPPS (SPSS Inc: Released 2021. SPSS for MacOS, Version 26 Chicago, SPSS Inc.).

### GVS Neuroimaging Experiment

Functional images were obtained in a clinical 3 Tesla MRI scanner (Skyra, Siemens, Germany) with a 64-channel array head and neck coil employing echo-planar imaging (EPI) with a fast T2* weighted gradient-echo sequence (TR= 500 ms, multi-band factor 6, 30 continuous axial slices covering the brainstem and the cerebellum, 2.5 mm isotropic voxels, field of view 210 mm^2^, TE= 34.8 ms, posterior-to-anterior phase encoding direction to minimize distortions in the brainstem regions of interest) modified for optimal infratentorial neuroimaging. A high-resolution T1-weighted MP-RAGE sequence was acquired in sagittal orientation (TR = 2060 ms\TE = 2.17 ms, flip angle = 12°, FoV = 240 mm, slice thickness = 0.75 mm, A-P phase encoding, 0.75 mm in-plane resolution, GRAPPA factor 2) for DARTEL-based normalization including geodesic shooting to MNI space during the subsequent preprocessing.

Participants were examined in a supine position with their eyes open. GVS was applied using a customized GVS stimulator after local anesthesia of the mastoid bilaterally to minimize side-effects of GVS. For the functional measurements, a randomized block design with a visual rest condition varying in length was used, in which subjects had to fixate a dot. The dot was projected using a monitor positioned behind the subjects’ head and an adjustable mirror box reflecting the patterns attached to the head coil at a viewing distance of 16 cm (field of view 30°×18.85°). Eye movements were recorded with an infrared VOG-unit (MRI-compatible camera, MRC systems, www.mrc-systems.de) after calibration. To reduce head movements, an inflatable head cushion (Crania Adult Pearltec, Schlieren, Switzerland) was used. The scanner room and bore were completely darkened. The participants wore earplugs.

### Tasks and Stimuli

Before the actual fMRI experiments, all participants were instructed outside the scanner. The fMRI experiment included a resting state session and two subsequent GVS sessions. During the resting state as well as during all GVS sessions, subjects had to fixate a dot at the center of the screen. The GVS sessions consisted of unilateral ramp stimuli applied on each mastoid (3mA) (block length 14 TR = 7s, 12 repetitions per stimulation for each side). During the second session, bilateral rectangular GVS stimuli were applied (block length 60 TR = 30 s, 5 repetitions for each side). The intertrial intervals consisted of a rest condition varying in length where subjects had to keep their eyes open and look straight ahead (16–28 TRs, 8–14 s). The GVS stimuli were chosen based on the results of the VOG experiment.

### Data Analysis for the Task-Based fMRI Sessions

A statistical analysis was performed using SPM12 (Version 7771, Wellcome Department of Cognitive Neurology, London, UK) running on MATLAB release 2019b (MathWorks, Natick, MA) after checking for session homogeneity and artifacts of the raw data. The images were realigned to the first one of each scanning session to correct for subject movement and were then normalized into the standard anatomical space defined by the Montreal Neurological Institute (MNI) template by means of the DARTEL algorithm including geodesic shooting using an existing MNI-template (http://nist.mni.mcgill.ca/?p=904) through the use of the CAT12 toolbox (version 1742) [28]. All stereotactic coordinates given in this paper therefore refer to the MNI coordinate system. The normalized images were smoothed with a three-dimensional isotropic 4 mm Gaussian kernel. A high-pass filter 225 s long and the realignment parameters were integrated into the design matrix. According to the general linear model, the effect of the different stimulation conditions on regional BOLD responses was estimated including the realignment parameters [29]. Statistical parametric maps (SPMs) were generated on a voxel-by-voxel basis with a hemodynamic model of the stimulation periods present during the session [30]. Single subject t-contrasts were computed for each stimulation condition compared with the rest condition. The GVS afternystagmus was modelled as events after GVS at the on-set of the afternystagmus. Linear parametric time modulation was investigated for the GVS and afternystagmus conditions. To test for effects on a between-subject basis, the condition images were entered into a second-level statistical analysis. Paired *t*-tests for the different GVS stimuli and the afternystagmus were performed using the linear t-contrasts. This approach corresponds to a random-effects analysis, extending the scope of inference to a larger population. Activation maps were considered significant at *p*<0.05 (TFCE, FDR corrected) after 10,000 permutations [31]. The results were localized and visualized using the anatomy toolbox [32], the Duvernoy’s atlas of the Human Brain Stem and Cerebellum [33], the SUIT toolbox template [34], and MRIcroGL by Chris Rhorden (https://www.mccauslandcenter.sc.edu/mricrogl/).

## Results

### Video-Oculography Recordings Outside the Scanner

**Fig. 2:**
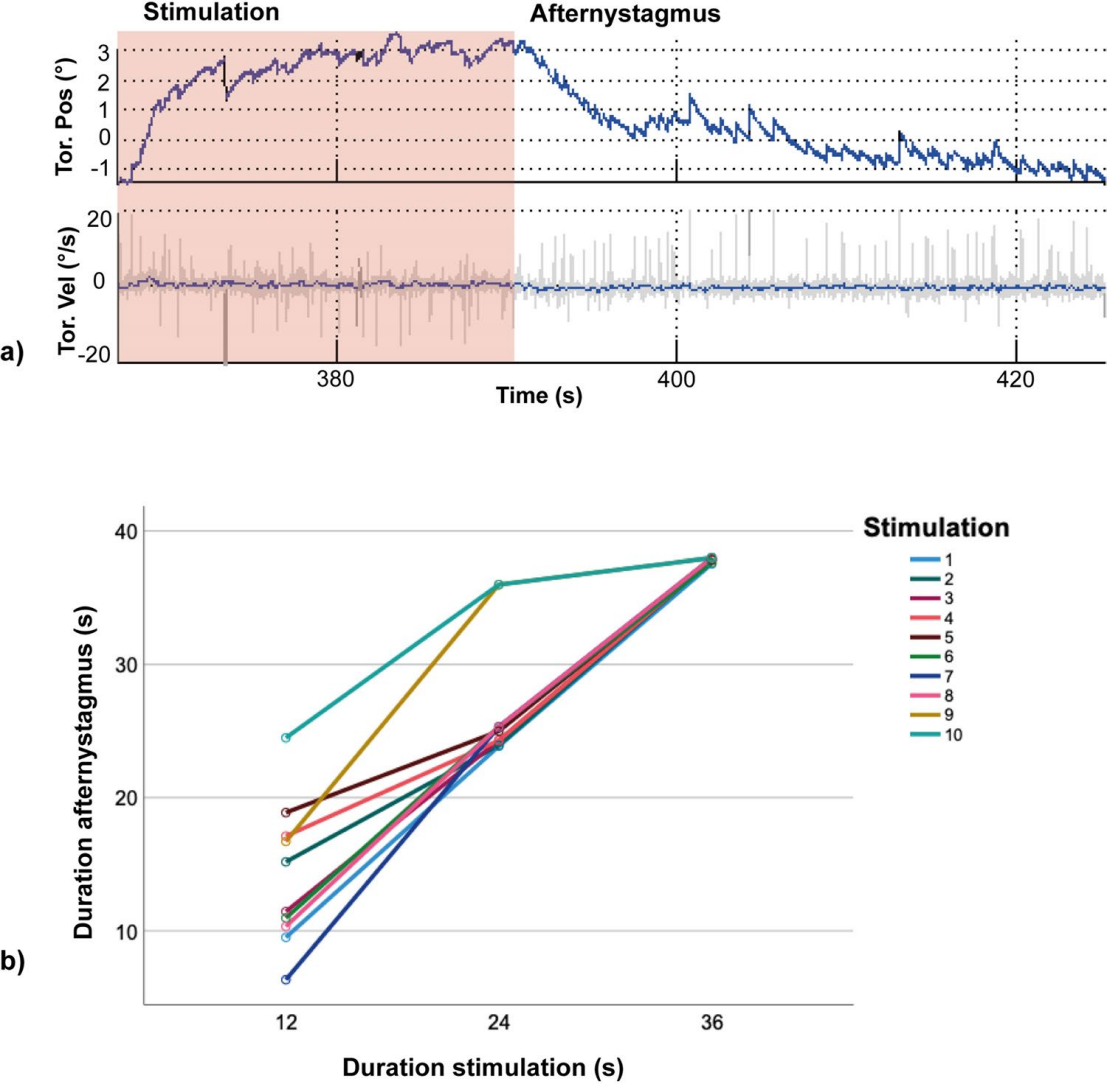
**Video-oculography findings for the GVS-induced afternystagmus.** (**a**) An exemplary sequence of video-oculography during a 36-s GVS stimulation (light red background) with the subsequent afternystagmus (white background). During GVS, apart from the torsional nystagmus, a tonic positional change of the eye was observed. At the offset of GVS, the nystagmus direction reversed and the tonic eye position deviation decayed back to baseline. (**b**) A display of the duration of the afternystagmus relative to the duration of the stimulation for each stimulation sequence from VOG experiment 2. The afternystagmus duration increased with increasing duration of GVS stimulation and increasing number of repetitions.

Our analysis was focused on the duration of the afternystagmus as a crucial parameter for the planning of the subsequent fMRI experiment. Time constants for the afternystagmus were not a goal in our experiments since they have already been studied extensively earlier [14, 21]. All subjects showed an afternystagmus with a predominant torsional component immediately at the off-set of GVS in both experiments. A mean torsional slow phase velocity of 0.30°/s (SD=0.10) after the 7-s ramp stimulus with 3 mA and a mean duration of 8.59 s (SD= 3.37) was observed in the first experiment. The torsional slow-phase velocity during stimulation (mean torsional slow-phase velocity = 0.79°/s, SD=0.96) was consistently higher than the afternystagmus velocity. To conduct a repeated measures analysis of variance (ANOVA) and be able to compare the responses of both stimulation sides regardless of their direction, responses in half the conditions were inverted. The repeated measure ANOVA revealed no statistically significant difference of the torsional slow-phase velocity or the duration of the afternystagmus with regard to the different intertrial intervals [6–24]. The slow-phase velocity of the nystagmus during and after stimulation did not significantly differ between left and right ear excitation. In VOG experiment 2, the rectangular GVS stimulus led to a mean slow-phase velocity of 0.43°/s (SD= 0.18). Significant differences of the afternystagmus with regard to the different stimulation durations [12, 24, 36] were observed in a repeated measure ANOVA (including Greenhouse-Geisser correction) *F*=3.12, *p*<0.05, partial *η*^2^ =.146 (see Fig. 2). No significant effect of stimulation duration on the induced torsional slow-phase velocity was found for the afternystagmus. The first afternystagmus was observed after a mean cumulative stimulation time (i.e., the mean of the summed up individual stimulation time in s for each subject until the first afternystagmus occurred) of 42 s with 3 mA current intensity (SD 41.5, minimum 12 s, maximum 132 s). After a mean cumulative stimulation time of 126 s with 3mA (SD 21.1, minimum 12 s, maximum 180 s), all subjects showed a robust afternystagmus persisting over the entire interstimulus interval of 38s and from then on occurring after every subsequent stimulation.

### fMRI Experiments

#### Infratentorial Responses During GVS with Rectangular Stimuli vs. Rest

**Fig. 3:**
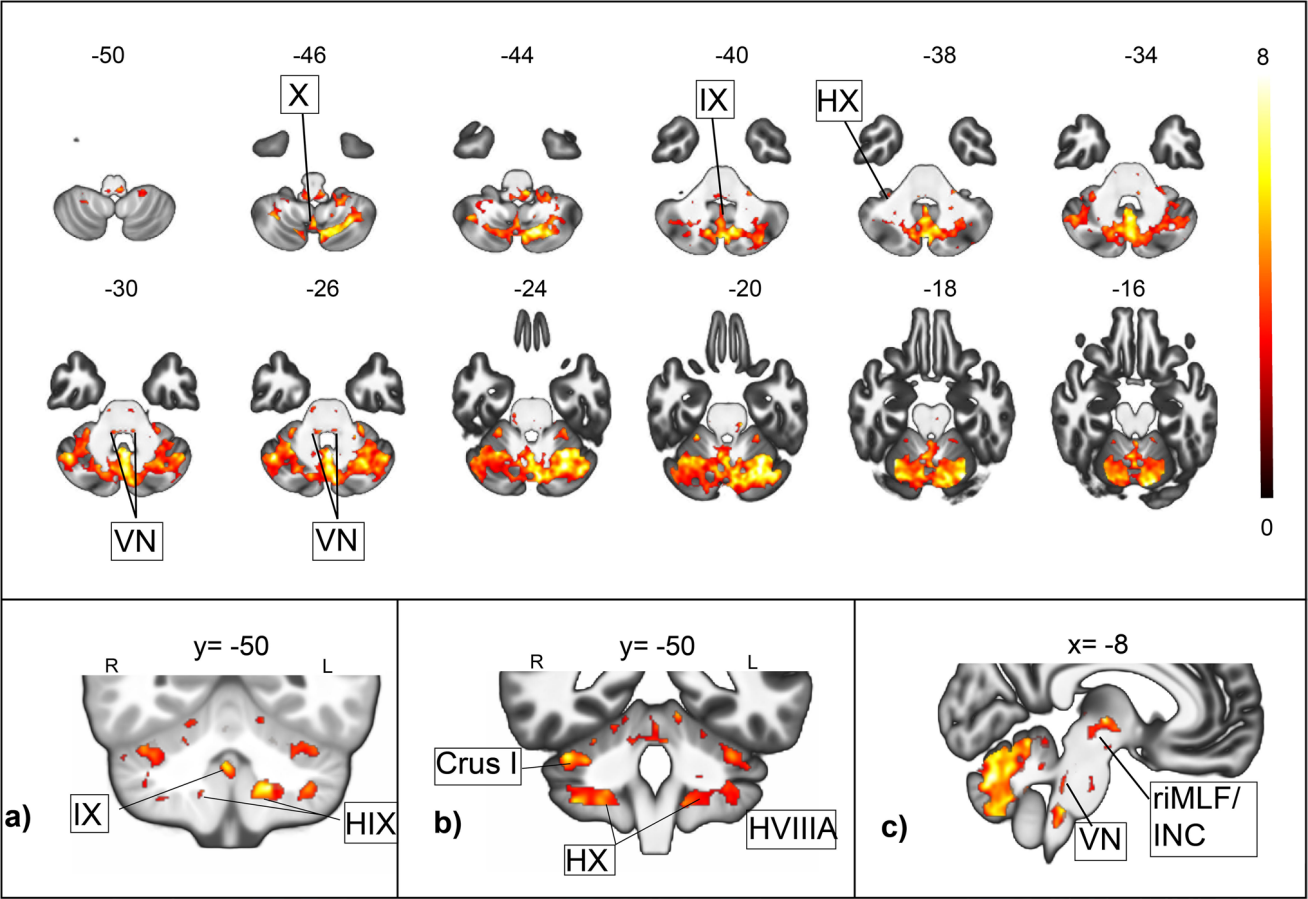
**Infratentorial responses to GVS.** The BOLD responses to the rectangular 30-s GVS stimulus in axial slices are depicted in the middle; the color scale on the right depicts *z*-scores. For a complementary visualization, the different vestibulocerebellar hubs are shown in coronal (top) and axial (bottom) slices as well. Top row: the rectangular 30-s GVS stimulus elicited response in the cerebellar vestibular hubs (**a**) (Uvula (IX), nodulus (not shown) and the cerebellar tonsils (HIX)) and (**b**) lobule HX (Flocculus) bilaterally, but also in Crus I, Crus II (not shown) and Lobule VIII bilaterally. Bottom row (**c**): significant responses were found for the vestibular nuclei (VN) bilaterally, apart from mesencephalic ocular motor regions (rostral interstitial nucleus of the medial longitudinal fasciculus (riMLF), interstitial nucleus of Cajal (INC)). All activation maps were thresholded at *p*< 0.05, FDR TFCE

The block and the ramp GVS stimuli elicited responses in the different cerebellar vestibular and ocular motor hubs, including the uvula, the nodulus, the flocculus and the tonsils bilaterally, and the dorsal ocular motor vermis, as well as Crus I and II and lobule VII and VIII bilaterally (Fig. 3). In the brainstem, responses were found in the vestibular nuclei bilaterally, as well as in mesencephalic ocular motor hubs as the rostral interstitial nucleus of the medial longitudinal fasciculus and the interstitial nucleus of Cajal (Fig. 3c). We found no significant differences between the ramp and the rectangular GVS stimulations for brainstem and cerebellar responses (all results are reported in detail in supplementary Table 1).

#### Activations for the GVS Afternystagmus Events

**Fig. 4:**
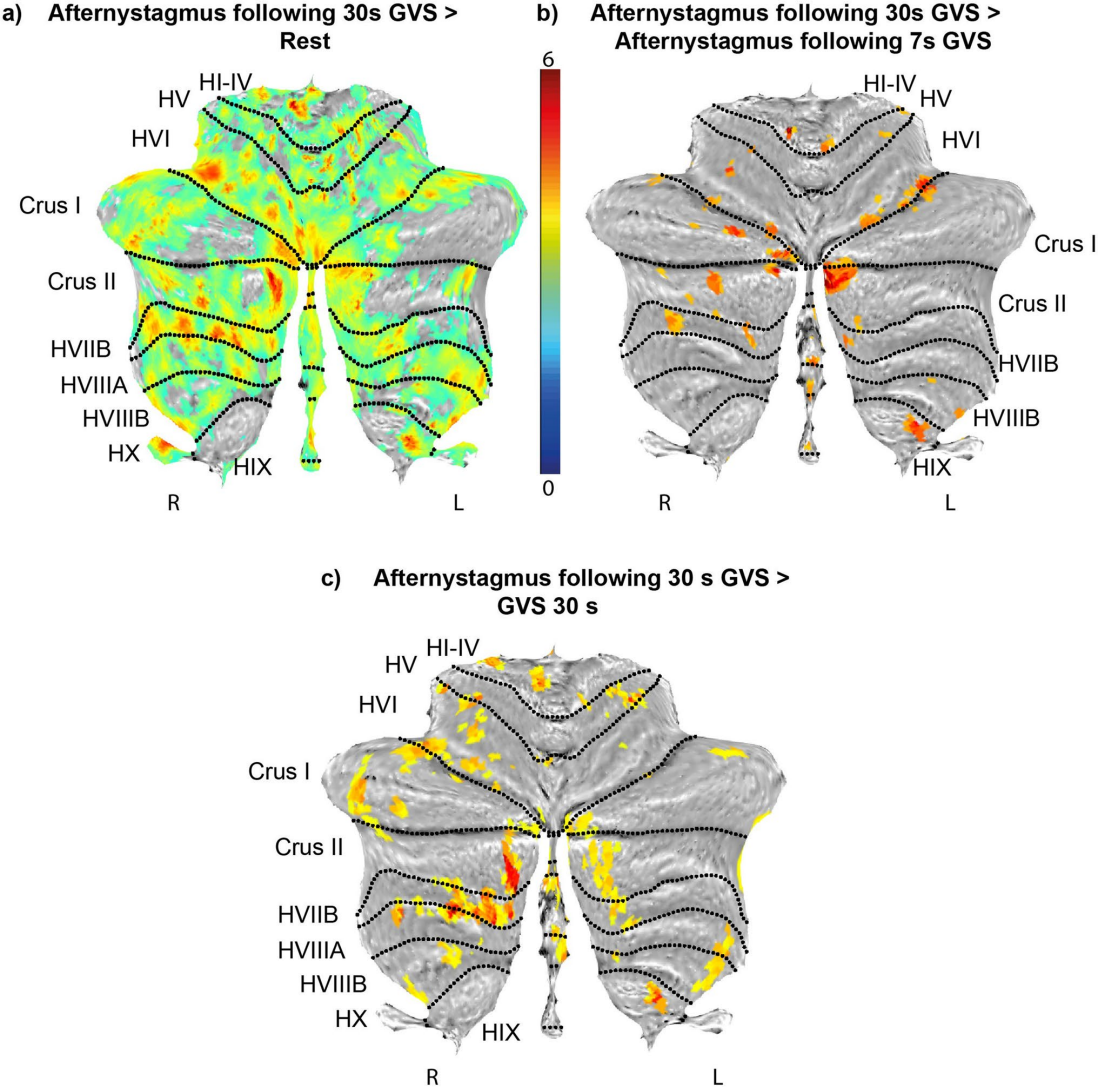
**Cerebellar responses during the afternystagmus.** Flatmap (**a**) shows the peak BOLD responses during afternystagmus subsequent to the 30-s rectangular GVS stimulation contrasted with the baseline condition (afternystagmus following 30-s GVS > Rest). Signal increased in the cerebellar hemispheres I-IV, VI, Crus I, II, VIIB the Flocculus and the left tonsil (HIX) as well as the uvula and nodulus, and in the dorsal ocular motor vermis. When contrasted with the afternystagmus following the shorter 7-s ramp GVS stimulus (section **b**) (afternystagmus following 30-s GVS> afternystagmus following 7-s GVS) and with the 30-s rectangular GVS stimulation (section **c**) (afternystagmus following 30-s GVS > GVS 30-s), peak responses appeared in Crus I-II, HVI, the left cerebellar tonsil (HIX) and vermal lobule VII, VIII and IX (uvula), hinting at a stronger involvement during the afternystagmus. The color scale depicts *z*-scores; all activation maps were thresholded at *p*< 0.05, FDR TFCE

**Fig. 5:**
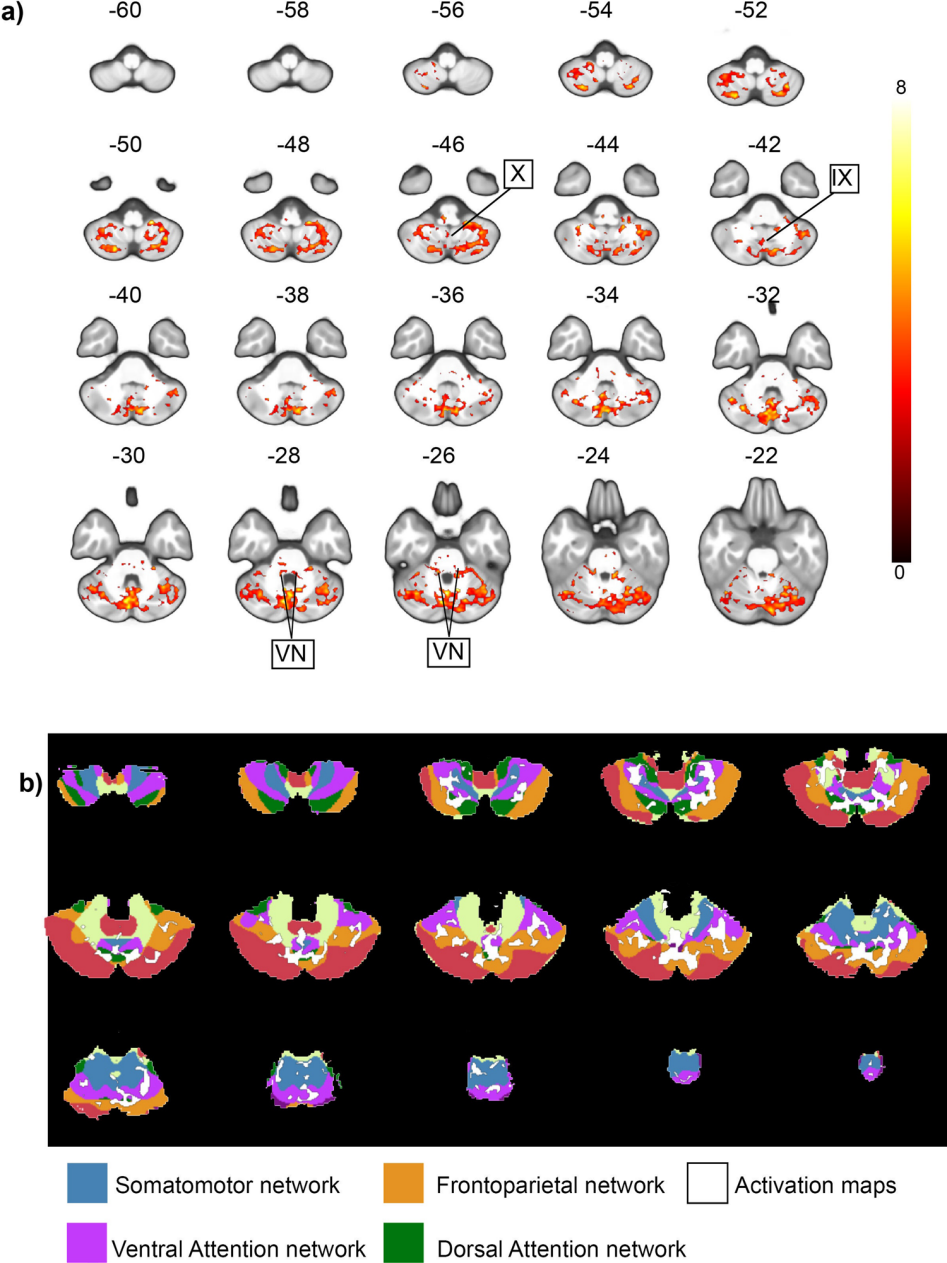
**BOLD responses during the GVS afternystagmus events.** Upper section (**a**) shows a slice view of the peak signal BOLD responses (red-yellow) during the afternystagmus following the 30-s rectangular GVS stimulus contrasted with the rest condition as correlate of the VSM and its cerebellar circuitries. Signal increases in the human vestibular nuclei (VN) correlate with evidence from animal studies localizing the VSM in the superior and medial VN. The activations in the vestibulocerebellar hubs in the vermis (uvula (lobule IX) and nodulus (lobule X)) correspond to their suggested inhibitory control function with regard to VSM reported in primates. The color scale on the right depicts *z*-scores. Section (**b**) shows an overlay of the activation maps (white) on a 7-network functional cerebellar parcellation after Buckner et al. [36]. Here, task-based activation maps overlapped with the ventral (pink) and dorsal (dark green) attention networks as well as the somatomotor network (blue) and the frontoparietal network (orange). All activation maps were thresholded at *p*< 0.05, FDR TFCE

The afternystagmus led to BOLD signal increases along the entire cerebellar vermis, with relevant cluster peaks in vermal lobule VIIIa and the uvula. Additional activations were localized bilaterally in the flocculus; Crus I and II; lobule VIIIa, VIIIb, and VI; and the left cerebellar tonsil. In the brainstem, responses were found in the vestibular nuclei. No significant habituation effects were found. When compared with established resting-state parcellations of the human cerebellum [35, 36], the delineated cerebellar activation maps coincide with the ventral and dorsal attention networks and frontoparietal networks and to a lesser extent with the somatomotor network (Figs. 4 and 5).

#### Afternystagmus vs. GVS Rectangular Stimuli

Contrasting the afternystagmus following 30-s-GVS stimulation block instead of 30-s GVS stimulation block gave significant increases in the uvula of the cerebellar vermis, vermal lobule VIIIa, the right flocculus, the cerebellar tonsils and lobule VIIIa and crus I bilaterally, and left lobule V, VI, and VIIb, Crus II, and the cerebellar dentate nucleus (Fig. 4).

#### Afternystagmus Following 30-s GVS vs. Afternystagmus Following 7-s GVS

We contrasted the afternystagmus after the two different stimulation conditions (30-s rectangular stimulation GVS vs 7-s ramp stimulation GVS) in order to depict the differences between the different velocity storage mechanism states. The afternystagmus after the 30-s rectangular stimuli conditions resulted in activations of the vermis (VIII and uvula), the right cerebellar tonsil, the right lobule VI, and Crus II bilaterally when contrasted with the aftereffects after the 7-s ramp stimuli (Fig. 4). No significant activations were found for the inverse contrast.

## Discussion

The neural substrates of the human VSM and its related circuitries were mapped in the vestibular nuclei and vestibulo-cerebellar hubs, but also in cerebellar regions far less investigated in relation to the human central vestibular system. Our results thereby emphasize the importance of cerebellar loops in VSM and give new insights regarding their function and embedment in the central vestibular circuitry of humans. GVS and tailored neuroimaging allowed for a precise localization of all vestibulo-cerebellar hubs known from non-human primates for the first time, including the uvula, nodulus, the flocculi, and the cerebellar tonsils, as well as the vestibular nuclei in the brainstem.

### Neuroanatomical Correlates of the Velocity Storage Integrator and Its Co-acting Mechanisms

For the first time, we could demonstrate structural correlates of the human velocity storage mechanism in vivo (Figs. 4 and 5). Our findings of an involvement of the vestibular nucleus are in full agreement with recent single unit recordings in non-human primates, suggesting that vestibular only neurons in medial and superior vestibular code for the velocity storage mechanism [37].

Moreover, our results stress the tight interaction between brainstem and cerebellum within the VSM network. Significant signal increases stretched along vestibulo-cerebellar regions in the cerebellar vermis including the uvula, nodulus, vermal lobule VIIIa, and the flocculus. These responses might represent inhibitory control circuitries acting on the VSM as proposed in the animal model [11]. Ablation of the uvula and the nodulus led to a prolonged velocity-storage time constant, whereas stimulation reduced the time constant [11, 38].Therefore, the uvula and nodulus are thought to be part of a “dumping mechanism” to discharge stored velocity and to dynamically modulate the time constant of the velocity storage mechanism, in particular when eye velocity exceeds surround velocity [39]. Their control of the time constants and cross-coupling parameters of the VSM have been hypothesized to be crucial for aligning the eye velocity towards the gravito-inertial acceleration [40–42]. In line with these findings, responses of the uvula, but also the vermal lobule VIII as well as the cerebellar tonsil were found in the contrast afternystagmus 30-s GVS vs. afternystagmus 8-s GVS and when contrasting the afternystagmus with GVS. This might indicate their predominant role in “discharging” the velocity storage mechanism (Fig. 5).

An involvement of vermal lobule VIII with the VSM has not been described in animal literature yet. In the macaque, though, projections from the medial vestibular nucleus, a crucial structure for the control of VSM, are known to reach lobule VIII [43]. Furthermore, a recent study in primates gave evidence for motor-cortex projections to vermal lobule VIII, hinting at a role in posture and locomotion control, which could be also confirmed in mice [44, 45]. In humans, responses of lobule VIII of the vermis could be shown during optokinetic nystagmus and saccades [46, 47]. To investigate the role of vermal lobule VIII with relation to vestibular processing and velocity storage in more detail remains an aim for future studies.

Apart from the vestibulo-cerebellar hubs established in animal literature, our results reveal bilateral responses in lobule VIIIa, and left lobule VIIIb and VIIb as well as Crus I and Crus II related to the VSM. These regions have so far not been associated with the VSM. In the animal model, projections from Crus I and Crus II to the vestibular nuclei have been described; nevertheless, their function in the human cerebello-vestibular system is not well investigated up to date [48]. Cerebellar Crus I, Crus II, and lobule VII are regarded as parts of the cerebellar nonmotor-network and recent studies could show an involvement of Crus I in different navigation tasks and spatial processing, including in the prediction of a position change over time based on visual speed information [49–52]. A recent work suggested a pathway of lobule VII, Crus I, and Crus II through the deep cerebellar nuclei to relay the cerebellum with navigation-related cells [53]. Furthermore, spatiotemporal tuning of optic flow responses and vestibular responses of Purkinje cells in the uvula and the nodulus in the macaque resemble those of area MSTd, hinting at a role in self-motion processing [54], for a role in Lobule VIII on the other hand is supposed to be part of the somatomotor network, with lobule VIIIB being in particular involved with somatosensory tasks [49]. At this point, we can only speculate in how far these areas might provide a link between vestibulo-cerebellar and spatial-navigation processing or are involved in feedback loops acting upon the VSM; nevertheless, our findings may pave the way for future investigations.

### Infratentorial Vestibular Activation Pattern by GVS

As a secondary result of our study, we mapped infratentorial responses to GVS with an unprecedented high temporal and spatial resolution, extending the present knowledge from the animal literature to humans. Although cortical response patterns to GVS have been studied to a large extent to date, the infratentorial responses have not been robust or localizable enough to be reported in detail. A few studies described activations of Crus I and in the vermis during GVS but did not report brainstem results [55, 56]. Corresponding to evidence from animal models indicating a recruitment of both hair cells and vestibular afferent fibers during GVS [16, 17], we were able to localize responses to GVS in the human vestibular nuclei (Fig. 3). Our findings of responses in the brainstem and cerebellar ocular motor regions like the riMLF and INC, as well as the dorsal ocular motor vermis, reflect the ocular motor activity during the task.

The signal increases in the uvula and nodulus correspond to findings in the animal literature indicating a central role in the integration of otolith and semicircular canal signals and determination of three-dimensional spatial orientation [40–42, 57]. Their reciprocal connections with the superior and medial vestibular nuclei and regions of the inferior olive involved in processing optokinetic and vestibular information have been demonstrated in different species [42, 58–60]. The floccular responses found during GVS correlate with its role in stabilizing gaze via inhibitory projections to the vestibular nuclei [61]. This might also explain the signal increases found in the cerebellar tonsils, although their exact function is unclear at this point. In animal literature their anatomical homologues, the dorsal paraflocculus and lobus petrosus, have been often regarded as a functional unit with the flocculus implying a role in eye-movement control, in particular smooth pursuit [62, 63]. Studies in rodents showed that the paraflocculus receives less visual and vestibular projections than the flocculus [64] and a study in monkeys suggested a role of its dorsal part in visuomotor coordination, relaying signals to cortical visual areas. In earlier studies we could show an involvement of the cerebellar tonsil optokinetic nystagmus and in physiological upbeat nystagmus, speaking for a role in gaze stabilization and in a possible “antigravitational’ pathway [27, 46, 65]. Our results here extend these findings and substantiate a role in visual-vestibular interactions. The responses found in Crus I, II, and VIII during GVS as well as during the afternystagmus are in line with lesion studies indicating a role in spatial executive function [66] and show their embedment in the vestibular infratentorial system.

### Video-Oculography Findings

In both experiments, all subjects showed an afternystagmus at the offset of GVS. In our study, the onset of the first afternystagmus varied among the subjects, possibly indicating a varying individual threshold. The few earlier studies mentioned above did not report on individual differences regarding this onset. One study in squirrel monkeys described a threshold depending OKAN [67]. In humans, so far there are no reports of threshold determination regarding the velocity storage mechanism to our knowledge. Using a high-intensity GVS stimulus with 5 mA and 5-min duration, Mac Dougall et al. could show a significant linear relationship of the GVS after-responses (ocular torsion position, torsional nystagmus), which could not be replicated using shorter stimulation times [14]. In agreement with another study using lower current intensities [22], using a 3-mA stimulus in our study elicited an afternystagmus with an expectedly lower slow phase velocity than the nystagmus during GVS (Fig. 2). None of the manipulations of intertrial interval or stimulus duration influenced the magnitude of eye movements in our experimental setup. This is also very much in line with the results of MacDougall [14]. In contrast to MacDougall et al. which showed a greater recovery of the afternystagmus with longer intertrial intervals after stimulation between 30 and 120 s, we did not find a significant effect of the duration of the intertrial interval on the duration of the afternystagmus. This might be explained by the often shorter stimulation times used in our study [6] which were targeted towards the ensuing neuroimaging experiment [14].

## Conclusion

In the present study, we demonstrated the structural correlates integral to the human velocity storage mechanism in humans in vivo. Our results emphasize the relevance of a cerebellar interaction with the vestibular nuclei within the human VSM network. We hypothesize that the cerebellar response found in the uvula and vermal lobule VIII might reflect inhibitory feedback loops corresponding to the proposed “dumping mechanism” of the VSM in the animal model. Adding up to the so far established cerebellar hubs interacting with the VSM in the nonhuman primate, we give direct evidence for an embedment of lobule VII and VIII, and Crus 1 and Crus 2 in the VSM and vestibular network. These findings thereby amplify the current functional understanding of these regions involved in sensorimotor processing (lobule VII) and spatial executive function (Crus I, Crus II, lobule VIII). As a secondary result, we successfully mapped the entire infratentorial vestibular system. We could demonstrate significant responses in all known vestibulo-cerebellar hubs and along the vestibular nuclei all of which had previously been established in non-human primates.

## Supplementary Information

Below is the link to the electronic supplementary material.

Supplementary file1 (DOCX 26 KB)

## Data Availability

Statistical group data and Matlab stimuli scripts are available upon reasonable request.
