# Supplementary material for: In Vivo Localization of the Human Velocity Storage Mechanism and Its Core Cerebellar Networks by Means of Galvanic-Vestibular Afternystagmus and fMRI
Source: Cerebellum. 2022 Feb 25;22(2):194–205. doi: 10.1007/s12311-022-01374-8 (PMC9985569; doi:10.1007/s12311-022-01374-8)
Supplement: Supplementary file 1 — Supplementary file1 (DOCX 26 KB) [file 12311_2022_1374_MOESM1_ESM.docx]

# Supplementary Table

| **Table 1**  **Results of the cluster peaks (t-contrasts) for all conditions**  (TFCE, FDR corrected, p< 0.05) | | | | | |
| --- | --- | --- | --- | --- | --- |
| **T-contrast** | **Brain area** | **cluster**  **size** | **t value** | **x, y, z** |  |
| **Afternystagmus> Baseline** |  |  |  |  |  |
| **Activations** |  |  |  |  |  |
|  | Cerebellum L VIIIa | 2229 | 8.82 | -26 -66 -54 |  |
|  | Cerebellum L Crus II |  | 7.52 | -8 -76 -38 |  |
|  | Cerebellum R VI |  | 7.03 | 6 -68 -28 |  |
|  | Cerebellum L Crus II |  | 7.52 | -8 -76 -38 |  |
|  | Cerebellar Vermis VIIIa |  | 6.85 | 4 -60 -30 |  |
|  | Cerebellum L VI |  | 6.74 | -24 -66 -18 |  |
|  | Cerebellar Vermis Crus II |  | 6.57 | 4 -74 -30 |  |
|  | Cerebellum Left X (Flocculus) |  | 6.39 | -15 – 42 -44 |  |
|  | Cerebellum Vermis IX (Uvula) |  | 5.43 | 4 -60 -36 |  |
|  | Cerebellum R VIIIa | 88 | 6.04 | 28 -44 -44 |  |
|  | Cerebellum R X (Flocculus) |  | 5.06 | 20 -42 -46 |  |
|  | Cerebellum R VIIIb |  | 4.11 | 20 -54 -50 |  |
|  | R Nucleus N. VIII |  |  | 7, -33, -26 |  |
|  | Cerebellum R Crus I | 30 | 6.46 | 46 -50 -30 |  |
|  | L Paramedian Pontine Reticular Formation (PPRF) | 29 | 6.90 | -6 -34 -30 |  |
|  | Vestibular nucleus |  | 6.8 | -5 -34 -31 |  |
|  | Cerebellum L VIIIb | 25 | 4.75 | -16 -54 -48 |  |
|  | Cerebellum L Crus I | 23 | 5.06 | 34 -64 -34 |  |
|  | Cerebellum L VI | 21 | 5.84 | -22 -62 -34 |  |
|  |  |  |  |  |  |
| **Afternystagmus > GVS Rectangular** |  |  |  |  |  |
| **Activations** |  |  |  |  |  |
|  | Cerebellum L VIIIa | 103 | 7.34 | 26 -66 -54 |  |
|  | Cerebellum L VIIb |  | 5.28 | 20 -70 -50 |  |
|  | L Vestibular Nucleus | 34 | 6.34 | -8 -34 -30 |  |
|  | Cerebellum L VI | 31 | 4.5 | 34 -54 -28 |  |
|  | Cerebellum L Crus II | 20 | 4.34 | -8 -76 -40 |  |
|  | Cerebellum L Crus I | 20 | 4.31 | -42 -48 -36 |  |
|  | Cerebellum R X (Flocculus) | 18 | 4.3 | 28 -42 -44 |  |
|  | Cerebellum L V | 18 | 5.12 | -8 -60 -26 |  |
|  | Cerebellum R Crus I | 17 | 4.10 | 14 -74 -38 |  |
|  | Cerbellar Vermis VIIIa | 10 | 4.48 | 0 -68 -44 |  |
|  | Cerebellar Vermis IX (Uvula) | 10 | 4.37 | 4 60 -36 |  |
|  | Cerebellum R IX (Tonsil) | 10 | 4.98 | 12 -48 -46 |  |
|  | Cerebellum R VIIIa | 10 | 4.04 | 22 -70 -54 |  |
|  | Cerebellar Dentate Nucleus | 10 | 4.53 | -22 -62 -34 |  |
|  | Cerebellar Left IX (Tonsil) | 5 | 3.95 | -18 -54 -48 |  |
| **Afternystagmus long (rectang.) > Afternystagmus short (ramp)** | Cerebellum R Crus II | 50 | 6.05 | 8 -78 -30 |  |
|  | Cerebellum L Crus II | 47 | 5.42 | -8 -82 -26 |  |
|  | Cerebellum R VI | 20 |  | 38 -58 -24 |  |
|  | Cerebellum Vermis VIII | 11 | 4.77 | 2 -68 -44 |  |
|  | Cerebellum L Crus II | 10 | 4.56 | -34 -56 -44 |  |
|  | Cerebellum R IX (Tonsil) | 8 | 5.16 | 12 -46 -48 |  |
|  | Cerebellum Vermis IX (Uvula) | 10 | 4.49 | 0 -56 -32 |  |
| **GVS Rectangular >baseline** |  |  |  |  |  |
| **Activations** |  |  |  |  |  |
|  | Cerebellum L Crus I | 14146 | 10.65 | -28 -74 -34 |  |
|  | Cerebellum L Crus II |  | 8.93 | -26 -76 -42 |  |
|  | Cerebellum R Crus I |  | 8.82 | 26 -76 -28 |  |
|  | Cerebellum Interposed Nucleus |  | 5.37 | 6 -56 -32 |  |
|  | Cerebellum L X (Flocculus) | 339 | 6.81 | -22 -38 -46 |  |
|  | Cerebellum L VIIIa |  | 5.03 | -32 -40 -52 |  |
|  | Cerebellum L IX (Tonsil) |  | 4.29 | -14 -48 -44 |  |
|  | Cerebellum R X (Flocculus) | 48 | 5.36 | 18 -32 -42 |  |
|  | Pons N. VIII (Nerve) | 38 | 4.54 | -8 -28 -44 |  |
|  | Pons Nucleus N. VIII |  | 3.75 | -6 -32 -36 |  |
|  | Cerbellum R VIIb | 38 | 6.83 | 20 -74 -48 |  |
|  | Cerebellum L VIIb | 10 | 5.58 | -34 -60 -48 |  |
| **GVS ramps > baseline** |  |  |  |  |  |
| **Activations** |  |  |  |  |  |
|  | Cerebellum L IX (Tonsil) | 6980 | 8.92 | -16 -50 -48 |  |
|  | Cerebellum L VIIIa |  | 8.19 | -10 -70 -48 |  |
|  | Cerebellum Vermis VI |  | 8.06 | 0 -68 -26 |  |
|  | Cerebllum L Crus II |  | 8.03 | -6 -76 -38 |  |
|  | Cerbellum L Crus I |  | 7.93 | -40 -54 -26 |  |
|  | Cerebellum L VI |  | 7.61 | -26 -64 -26 |  |
|  | Cerebellum L X (Flocculus) |  | 7.5 | -22 -36 -46 |  |
|  | Cerebellum L V | 18 | 4.9 | 10 -54 -14 |  |
